# Pilot study of a local exhaust ventilation system to control particle and terpene concentrations during cannabis processing

**DOI:** 10.3389/fpubh.2026.1964267

**Published:** 2026-10-23

**Authors:** Callan Krevanko, Charlotte Beatson, Martin Cohen, Coralynn Sack, Christopher D. Simpson

**Affiliations:** 1 Department of Environmental and Occupational Health Sciences, University of Washington, School of Public Health, Seattle, WA, United States; 2 Department of Medicine, University of Washington, Seattle, WA, United States

**Keywords:** cannabis, engineering controls, grinding, industrial hygiene, joint filling, LEV, particulate matter, VOCs

## Abstract

**Introduction:**

With the expansion of the U. S. legal cannabis market, there is increased awareness of health and safety concerns in the cannabis industry. Recent evidence suggests a growing burden of health symptoms in cannabis production workers, who may be exposed to toxic agents, including respiratory irritants and allergic sensitizers, during their job activities. Swift action is warranted to protect this expanding workforce by characterizing and controlling airborne exposures during cannabis production.

**Methods:**

To help address these concerns, we developed a local exhaust ventilation (LEV) system with a partially-enclosed hood to control particle and terpene emissions during cannabis processing activities using a minimum efficiency reporting value (MERV)-13 filter (particles) and an activated charcoal filter (terpenes). Inhalable particulate matter (PM) and total terpene air concentrations were compared with and without the LEV system across three separate paired trials during whole flower grinding and automated pre-roll joint filling.

**Results:**

On average, the LEV system reduced inhalable PM concentrations by 95% during whole flower grinding and 83% during pre-roll joint filling. The LEV system was similarly effective in controlling total terpenes, reducing concentrations to below the limit of detection (LOD) during whole flower grinding.

**Discussion:**

To date, this is the first study to evaluate the efficacy of LEV as an exposure control during cannabis processing. These preliminary results suggest that the partially-enclosed hood LEV system is an effective exposure control for cannabis processing activities. Findings from this study can guide future industrial hygiene interventions that protect cannabis workers from potentially harmful exposures.

## 1 Introduction

Washington and Colorado became the first U.S. states to legalize recreational cannabis use in 2012, with commercial sales commencing in 2014 (1, 2). Since then, the number of full-time jobs supported by the U. S. legal cannabis industry has grown quickly, supporting an approximated 425,000 workers by 2025 (3). The rapid expansion of the legal cannabis industry has prompted increased awareness of occupational health and safety concerns during cannabis production. Multiple cases of work-related asthma (WRA) have been identified in cannabis processing workers (4), including at least two fatalities that were attributed to cannabis dust exposure (5, 6). Other recent studies have also elicited concern about allergic and respiratory health effects attributed to cannabis processing exposures (7–11), although the exact cause of these symptoms remains unknown.

The first phase of cannabis production is the cultivation of cannabis plants– a process optimized by hot and humid conditions in either indoor or outdoor environments (12). After the mature cannabis plants are harvested and dried, workers process dried cannabis through a series of steps, including the removal of leaves and flowers from the primary plant stem; the manual or mechanical trimming of cannabis flower; the grinding of cannabis flower for joint material; and the filling of pre-roll joints (13–17). Additionally, the ground cannabis material may be extracted with a variety of solvents to generate cannabinoid-rich concentrates which are used in a wide variety of consumer products. During each processing task, workers are potentially exposed to respiratory irritants and allergic sensitizers, including cannabis dust, microbial contaminants, pesticides, and VOCs, including terpenes (18–21). Terpenes can readily autoxidize under ambient conditions to form byproducts that may induce allergic sensitization (22–25). Although few studies have characterized these exposures across cannabis production tasks, preliminary evidence has found elevated airborne levels of several of these agents during trimming, joint filling, and grinding (17, 19, 26).

The emerging evidence of work-related health symptoms in cannabis production workers underscores the need to better characterize exposures during cannabis processing. Given the severity of reported health effects—including two reported asthma fatalities—there is also an urgent need for exposure controls, even with the remaining uncertainties about workplace exposures. The implementation of uniform exposure controls may be challenging in the cannabis industry, where there is substantial heterogeneity in facility size, building infrastructure, production practices, and ventilation systems (7, 17, 19, 27, 28) [(11), *submitted*]. Indoor production frequently occurs in retrofitted warehouses (7). In some facilities, ventilation is intentionally suppressed to maintain optimal growing conditions and reduce cannabis odors outside the facility (19, 20, 27). Task-level controls may therefore be a practical option because they can be integrated into existing operations without requiring major changes to facility infrastructure. Local exhaust ventilation (LEV) has been recommended as an exposure control for cannabis processing tasks (13, 29), but to date, no studies have evaluated its effectiveness in reducing occupational exposures in this industry. To address this need, we conducted a laboratory-based pilot study to design and evaluate a partially-enclosed hood LEV system to control breathing zone concentrations of particulate matter (PM) and terpenes during cannabis processing activities, including automated pre-roll joint filling and whole flower grinding.

## 2 Materials and methods

### 2.1 System design

We designed an LEV system to reduce PM concentrations during the grinding of cannabis flower and the automated filling of pre-roll joints with ground cannabis. The system, depicted in Figure 1, featured a partially-enclosed hood that surrounded the cannabis grinder or joint filler (“knockbox”), with the open face oriented toward the worker. While a fully-enclosed hood system was initially evaluated, preliminary results from laboratory and field-based testing suggested that the design was ineffective because workers frequently needed to open the enclosure. A partially-enclosed hood was therefore selected in lieu of a fully-enclosed hood to allow workers to easily maneuver the system and manually manipulate equipment and materials. A 4”x20”x20” air filter [minimum efficiency reporting value (MERV)-13] was attached to the back of the partially-enclosed hood to control particle emissions. Airflow through the system was maintained using a VIVOSUN AeroZesh S8 8” in-line mixed flow fan (Ontario, CA USA). During grinding experiments, the system was additionally equipped with an annular 8” internal diameter activated charcoal filter (VIVOSUN–Ontario, CA USA) downstream of the fan to capture VOC terpenes. The charcoal filter was used during grinding but not joint filling trials because prior research suggests that VOC terpene levels are especially elevated during cannabis grinding but not as high during pre-roll joint filling (26).

**Figure 1:**
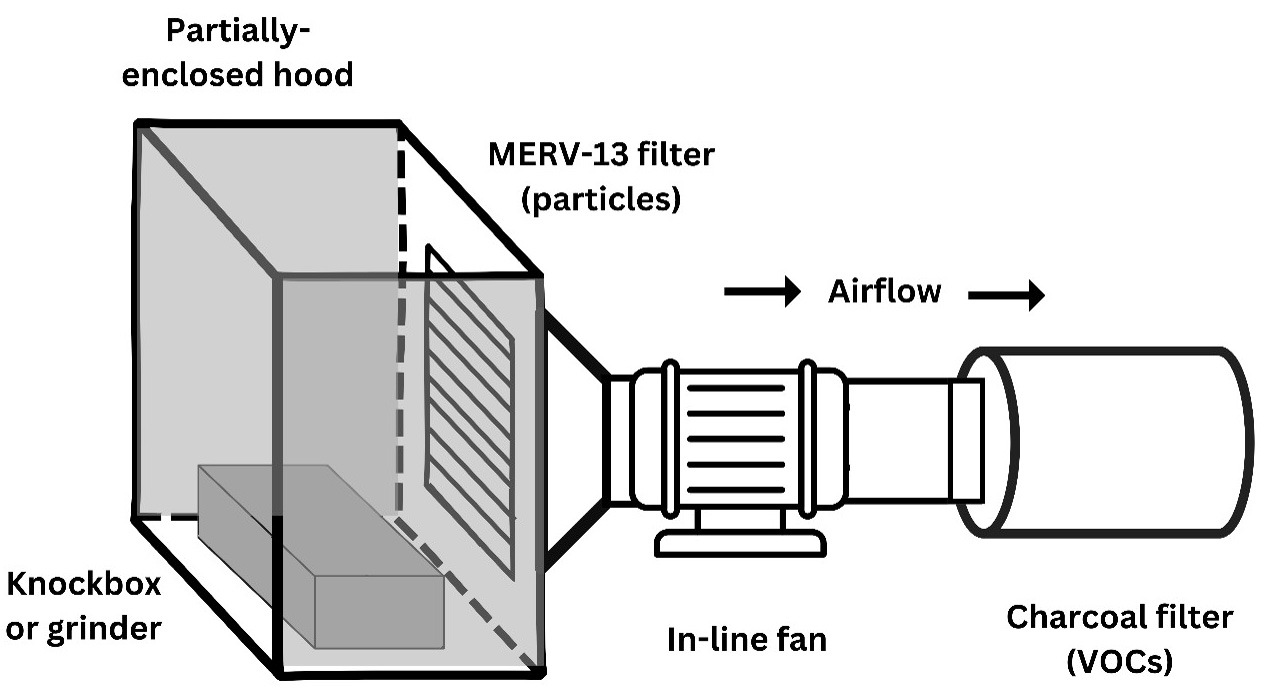
Schematic of the LEV partially-enclosed hood system for exposure control during cannabis grinding and automated joint filling. For cannabis grinding experiments, the charcoal filter was installed downstream of the filter (as shown) for control of terpene emissions. For joint filling experiments, the charcoal filter was not used.

The average face velocity and airflow for each filter configuration were determined using a hot wire anemometer based on a 16-point traverse (Table 1). For select trials, an aerodynamic particle sizer (APS) (Model 3321; TSI Inc.–Shoreview, MN) characterized the mass aerodynamic particle size distributions during cannabis production activities. APS data from grinding and joint filling trials are available in the Supplementary Materials.

**Table 1:** Overview of experimental activities and conditions.

Activity	Filter(s)	LEV face velocity (ft/min)	LEV airflow (CFM)	Analytes measured
Joint filling	MERV-13 (particles)	231	924	inhalable PM (gravimetric); total PM (continuous)
Whole flower grinding	MERV-13 (particles); activated charcoal (terpenes)	198	792	inhalable PM (gravimetric); total PM (continuous); total terpenes

CFM, cubic feet per min; MERV, minimum efficiency reporting value; PM, particulate matter.

### 2.2 Whole flower grinding

Three separate laboratory-based paired trials (with LEV vs. without LEV) were conducted to assess the efficacy of the partially-enclosed hood system in controlling PM and terpene concentrations during cannabis grinding. For each paired trial, the gravimetric inhalable PM, continuous total PM, and total terpene air concentrations were compared with and without the LEV system during the coarse grinding of whole cannabis flower (~340 g) over approximately ten min. Cannabis flower was ground using the Wacky Tobacky Shredder commercial grinder (Wacky Willy's–Victoria, Canada). This grinder is designed for small to medium sized commercial operations. Cannabis (hemp) containing < 0.3% tetrahydrocannabinol (THC), was used due to US federal restrictions on possession and use of cannabis with >0.3% THC.

### 2.3 Joint filling

Three separate laboratory-based paired trials were also conducted to assess the efficacy of the partially-enclosed hood system in controlling PM concentrations during joint filling. For each paired trial, gravimetric inhalable PM and continuous total PM concentrations were compared with and without the LEV system during the automated filling of 800 pre-roll cones over approximately 75 min. The pre-roll cones were filled with ground hemp generated from the grinding experiments described above using the SnowDraft knockbox (Archibold–Dongguan, China), which simultaneously fills 100 pre-roll cones with one gram of coarsely ground cannabis each in approximately two min. This device is comparable to other commercially used models in both function and production volume.

### 2.4 Measurement of particulate matter and VOC concentrations

Static sampling of PM and VOCs was undertaken with all sampler inlets located in front of the LEV system, in the approximate location of the breathing zone of the operator of the grinder or knockbox. For grinding and joint filling trials, gravimetric inhalable PM samples were collected with the IOM inhalable dust sampler (SKC–Eighty Four, PA USA) equipped with a 25mm diameter, 5 μm pore size PVC filter. The IOM sampler was affixed to a GilAir Gilian Plus sampling pump (Sensidyne–St. Petersburg, FL USA), which was calibrated to 2.0 liters per min (LPM) using the Defender 510 DryCal calibrator (MesaLabs–Lakewood, CO USA). Filters were handled according to NIOSH Method 0500 (30) and weighed on a XS3DU microbalance (Mettler Toledo–Columbus, OH USA) in an AIHA-accredited laboratory. Continuous total PM concentrations were measured using the Personal DataRam pDR-1500 active Personal Particulate Monitor (pDR; total PM inlet; Thermo Scientific–Waltham, MA USA), which was zeroed prior to sampling according to the manufacturer specifications. After sampling, total PM measurements collected by the pDR were calibrated using a gravimetric sample collected on an internal filter. Due to spatial constraints, the pDR was located approximately six inches farther from the knockbox or grinder than the IOM, but still within approximate location of the operator breathing zone.

During grinding trials only, total terpene air concentrations were sampled using glass Carbotrap T420 thermal desorption tubes that were specifically developed for measurement of terpenes (Supelco, Inc.–Bellefonte, PA USA) (31) and mounted in the TDS^3^ storage and sampling container (Supelco, Inc.–Bellefonte, PA USA). The thermal desorption tube was connected to the same GilAir pump as the IOM sampler using a dual-flow manifold (Sensidyne LP, St Petersburg, Fl, USA). The pump was set to run in constant high-pressure mode (20” H_2_O), with flow through the thermal desorption tube set to 0.05 LPM. Flow through the thermal desorption tube was calibrated with a Defender 510 DryCal calibrator (MesaLabs–Lakewood, CO USA) prior to sample collection and verified after sample collection. After sampling, thermal desorption tubes were stored at 4C° before shipping overnight on blue ice to an independent laboratory for analysis of 31 terpenes (see Supplemental Table 2) using gas chromatography-mass spectrometry (GC-MS). The GC-MS analysis was performed using a Restek Rxi-624Sil MS column (30m x 0.25mm ID, 1.4 μm film thickness). The GC temperature was held at 45 °C for two min; increased to 120 °C at a rate of 10 °C/min and held for three min; and finally increased to 280 °C at a rate of 18 °C/min and held for four min. Mass detection was performed using electron ionization at 70 eV with a full scan from *m/z* 40–265. The source temperature was 220 °C and the interface temperature was 250 °C. Calibration standards were prepared using Terpene Mix A and B (Supelco, Inc.–Bellefonte, PA USA) and myrcene (Sigma-Aldrich–Burlington, MA USA). Calibration levels ranged from 40 to 8,000 ng.

Paired trials were consecutively conducted on the same day and in the same controlled laboratory environment to minimize environmental differences that could confound results. Within paired trials, the same hemp batch was used to limit potential differences related to dryness or cultivar. Across paired trials, the sampling order alternated between starting with or without the LEV system to minimize any potential impacts related to the temporal sequence of sampling events. Field blanks for PM and terpenes were prepared and handled alongside samples during each paired trial. Field blanks were assembled in the same sampling apparatus as samples, but no air was drawn through them. Following sampling, field blanks were stored, handled, and analyzed in the same manner as samples.

### 2.5 Data analyses

Inhalable PM concentrations were computed according to NIOSH method 0500 (30) using the blank-adjusted filter masses and the average flow rates determined by pre- and post-sampling flow measurements. Continuous total PM concentrations were plotted against time to visually assess the effect of the LEV system on PM concentrations. Total detected terpene concentrations were calculated by summing the mass of quantifiable terpenes for each sample and dividing by the sample volume. ß-Myrcene was not included in the calculation of total terpenes because it did not meet the independent laboratory's internal quality control accuracy criteria. No terpenes were detected in any field blank, therefore no blank adjustment was necessary. For gravimetric samples, LODs were computed by dividing the average field blank mass change by the sample volume. For total terpene samples, LODs were computed by dividing the average mass analytical LOD (Supplemental Table 2) by the sample volume. Measurements below the LOD were substituted with LOD/2. All analyses were performed using RStudio Version 2025.05.1 (Posit Software, PBC).

## 3 Results

### 3.1 Whole flower grinding

During whole cannabis flower grinding, inhalable PM concentrations ranged from 2.4 to 8.2 mg/m^3^ (arithmetic mean [AM]: 5.3 mg/m^3^, standard deviation [SD]: 2.9 mg/m^3^) without the LEV system and were below the LOD (< 0.093 mg/m^3^ and < 0.12 mg/m^3^) to 0.68 mg/m^3^ with the LEV system (Table 2A). In two of the three grinding trials, the LEV system attenuated inhalable PM concentrations by approximately 99%, such that they were below the LOD after blank correction. In the third grinding trial, the LEV system was associated with a 72% reduction in inhalable PM concentration. Without the LEV system in place, the continuous total PM time series exhibited numerous concentration spikes during grinding; these spikes, which were likely associated with frequent re-loading of whole cannabis flower into the grinder, were not observed with the LEV system (Figure 2A).

**Table 2A:** Change in gravimetric inhalable PM and total terpene concentrations with LEV during cannabis flower grinding.

Paired trial	LEV status	Inhalable PM concentration (mg/m^3^)^*^	% Change with LEV
TRIAL 1	LEV	< 0.093	−99%
	No LEV	8.2	
TRIAL 2	LEV	< 0.12	−99%
	No LEV	5.2	
TRIAL 3	LEV	0.68	−72%
	No LEV	2.4	
AVERAGE	LEV	0.26 (SD: 0.36)	−95%
	No LEV	5.3 (SD: 2.9)	
Paired trial	LEV status	Total terpene concentration (mg/m^3^)	% Change with LEV^**^
TRIAL 1	LEV	< 0.084	−96%
	No LEV	1.1	
TRIAL 2	LEV	< 0.14	−97%
	No LEV	2.6	
TRIAL 3	LEV	< 0.11	N/A
	No LEV	< 0.11	

LEV, Local exhaust ventilation; PM, particulate matter; SD, standard deviation.

^*^LODs were calculated by taking the average of blank mass changes (PM) or the weighted average of mass LODs (terpenes) and dividing by sample volume. For paired trials including measurements < LOD, the percent change and average concentration across trials were calculated by substituting LOD/2.

^**^No terpene concentrations were detected during the third grinding trial, therefore no percent change is presented. The absence of detectable terpene concentrations without the LEV system during this trial may be due to hemp batch variation or terpene volatilization prior to the experiment. No average percent change with LEV is presented for total detectable terpene concentrations due to the limited number of samples with detectable terpene masses.

**Figure 2:**
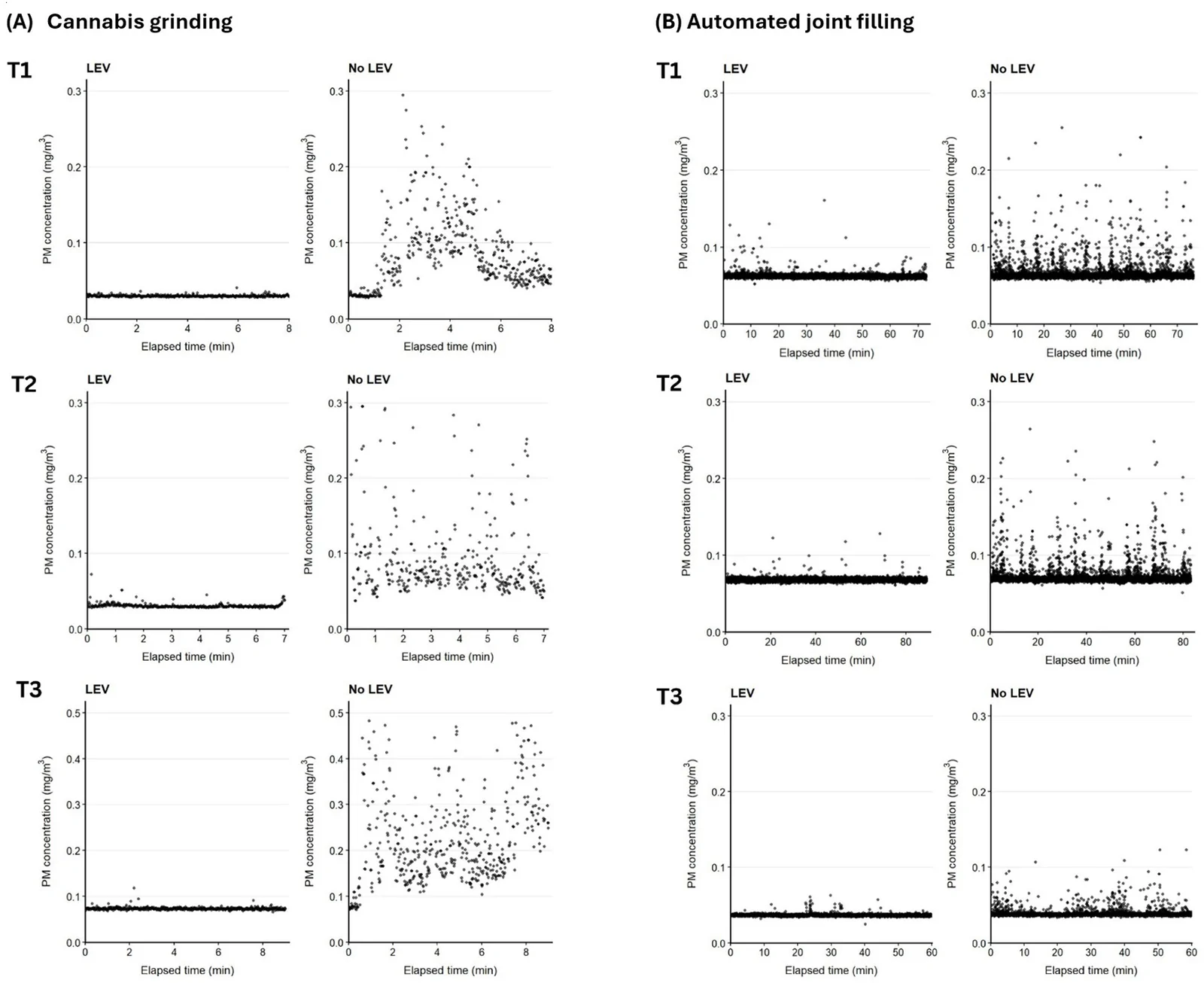
Continuous total particulate matter (PM) concentrations with and without the local exhaust ventilation (LEV) system across three paired trials (T1–T3) during **(A)** whole flower cannabis and **(B)** joint filling.

Six terpenes were detected across all three grinding trials: α-pinene, ß-pinene, ß-myrcene, p-cymene, d-limonene, and terpinolene. In two of the three grinding trials, the total detected terpene concentrations were reduced to below the LOD (< 0.084 mg/m^3^ and < 0.14 mg/m^3^, respectively) by the LEV system. Terpenes were not detected in the third trial under either ventilation condition, therefore no percent reduction associated with the LEV system was calculated.

### 3.2 Joint filling

Across automated joint filling trials, inhalable PM concentrations ranged from 0.080 to 0.25 mg/m^3^ (AM: 0.16 mg/m^3^, SD: 0.085 mg/m^3^) without the LEV system and 0.0045 to 0.049 mg/m^3^ (AM: 0.027 mg/m^3^, SD: 0.022 mg/m^3^) with the LEV system (Table 2B). On average, the LEV system reduced inhalable PM concentrations by approximately 83%. Continuous total PM concentrations additionally demonstrated strong reductions with the LEV system, with concentration peaks in the timeseries of PM measurements almost completely suppressed by the LEV system (Figure 2B).

**Table 2B:** Change in gravimetric inhalable PM concentrations with LEV during joint filling.

Paired trial	LEV status	Inhalable PM concentration (mg/m^3^)	% Change with LEV
TRIAL 1	LEV	0.0045	−98%
	No LEV	0.25	
TRIAL 2	LEV	0.027	−66%
	No LEV	0.080	
TRIAL 3	LEV	0.049	−68%
	No LEV	0.15	
AVERAGE	LEV	0.027 (SD: 0.022)	−83%
	No LEV	0.16 (SD: 0.085)	

LEV, Local exhaust ventilation; PM, particulate matter; SD, standard deviation.

## 4 Discussion

These pilot results suggest that the partially-enclosed hood LEV system is highly effective in reducing PM concentrations during cannabis grinding and joint filling. With the LEV system in place, strong reductions in inhalable PM were observed across all grinding and joint filling trials. Although limited terpene data was collected, these preliminary findings suggest that the LEV system is also effective in attenuating terpene concentrations during whole flower grinding.

Across all trials, inhalable PM levels were below the American Conference of Governmental Industrial Hygienists (ACGIH) recommended exposure limit for inhalable dust (10 mg/m^3^) (32). There was some heterogeneity in inhalable PM concentrations across paired trials, which may be related to varied hemp dryness, as different batches were used across (but not within) paired trials. To date, few studies have characterized PM exposures during cannabis production tasks and no studies have evaluated inhalable PM exposures during joint filling or grinding activities. However, recent health hazard evaluations (HHE) of cannabis grow facilities by National Institute for Occupational Safety and Health (NIOSH) have reported total PM concentrations during similar cannabis production tasks. In one recent HHE, NIOSH measured continuous total PM concentrations (60-s logging intervals) during dry cannabis processing tasks, including pre-roll joint production, sorting/weighing cannabis flower, and bucking dried cannabis plants. Average total PM concentrations ranged from 0.023 mg/m^3^ to 0.123 mg/m^3^ across these work areas. In another HHE at a separate facility, NIOSH investigators found that the average continuous total PM concentration (60-s logging intervals) was 3.4 mg/m^3^ during cannabis grinding (13). Similarly, Silvey et al. (26) found the average total PM concentration to be 0.045 mg/m^3^ during pre-roll joint filling at two indoor cannabis production facilities in Washington state. Our own recent studies in cannabis producers and processors in Washington state observed full-shift inhalable PM exposures between 0.2 and 77 mg/m^3^ (36). These studies are not directly comparable to the present analysis because of differences in the PM mass fractions measured, sampling methods, facility characteristics, and room ventilation characteristics. Instead, these concentrations are presented to provide context on the magnitude of PM concentrations generated in our laboratory-based experiments, which were within the range of concentrations previously measured in cannabis workplaces.

Although PM concentrations associated with cannabis production are typically below the OSHA permissible exposure limit (PEL) for particulates not otherwise regulated (PNOR), emerging evidence suggests that airborne exposures during cannabis processing activities may still pose an inhalation hazard (16). A recent paper reported 30 cases of work-related asthma in cannabis industry workers across four U. S. states, all of which were identified following legalization of recreational cannabis use in those states. The authors noted cannabis dust to be the most frequently reported exposure among cases (6). Other studies have also implicated cannabis processing exposures in the development of work-related asthma (4, 8, 10) and other allergic or respiratory health symptoms (7, 9, 11). While the cause of these health effects is not fully understood, growing evidence suggests that allergic sensitization to cannabis plant material may be an underlying mechanism of disease (16). Consequently, current occupational exposure limits based on generic particles may not be sufficiently protective against cannabis dust, as sensitized workers may experience health effects at lower mass concentrations (16, 21). Taken together, the research underscores the urgent need for the development and evaluation of exposure controls during cannabis processing activities. To our knowledge, this is the first study to assess LEV as an exposure control during cannabis production.

An important strength of this study was its ability to control many factors that could potentially confound results by influencing PM concentrations. The paired design enabled comparisons between the presence and absence of the LEV system within the same timeframe, space, and environmental conditions. In paired experiments under laboratory conditions, work practices mimicked those used by cannabis processors and were designed after consultation with industry partners and observations of cannabis production facilities. The methodical system design and evaluation suggest that this LEV system could be an economical and adaptable approach that could be adopted by cannabis producers and processors. The LEV system is easy to assemble and uses primarily off-the-shelf components with a total cost of approximately $500, making it economically and technologically feasible for use by small- and medium-sized businesses. The cost of ongoing operation and maintenance was not evaluated in this study and will depend on the use life of the filters, which should be assessed in future studies.

While these pilot findings support the strong potential benefits of the LEV system in cannabis production settings, the generalizability of results is limited because all tests were conducted under controlled laboratory conditions. Although the system was carefully designed with its end-use in mind, field-based testing is ultimately needed to fully assess its feasibility and efficacy in the workplace, and whether it reduces occupational health effects. Additionally, this pilot study included only three experimental trials, which limited our ability to characterize the variability in LEV performance and the generalizability of our findings. However, substantial reductions in breathing zone concentrations were consistently observed across all trials, providing preliminary proof-of-concept evidence that supports further evaluation of this LEV design under field conditions. Another key study limitation is that the LEV system was tested with just one style of grinder. There is substantial heterogeneity in work practices within the cannabis industry (20), with equipment and processes varying according to facility size and production volume. While the grinder used in this study is an example of some in use at small- to medium-sized facilities, it does not necessarily represent the grinders used across all facilities. Although the open-face design of the LEV system provides flexibility for use with different grinder models, it is unknown how effective the ventilation is for grinders with a higher throughput or different blade configuration. Additional testing with other equipment styles would therefore strengthen the generalizability of findings. We were also limited in our ability to assess terpene exposure control, as detectable terpene levels were observed in just two samples. Reducing terpene air concentrations may be important in cannabis production settings because it could limit the formation of potentially sensitizing terpene oxidation products. Although terpene exposure control could not be fully assessed in these trials, the limited findings suggest that the activated charcoal filter can effectively reduce terpene air concentrations during cannabis grinding. This finding is supported by another recent study that demonstrated reduced terpene air concentrations with use of a similar activated carbon filter (33). Although the charcoal filter was not used during joint filling trials, the strong exposure reductions observed during grinding suggest that the system could also effectively filter terpenes during joint filling, which is typically associated with lower terpene air concentrations than grinding (26). Further testing is recommended to assess the use life of the carbon filter and to determine how workers can assess filter saturation. Finally, we necessarily used hemp for these experiments due to federal restrictions on cannabis containing >0.3% THC. While THC content is not expected to meaningfully affect particle generation, there is some evidence that suggests that cannabinoid chemotype may be associated with different terpene profiles (34, 35). Future testing in cannabis workplaces can help determine whether differences in cannabinoid content influence the LEV performance.

Even with the noted limitations, these pilot study findings suggest that a partially-enclosed hood LEV system can effectively control particle emissions during cannabis processing tasks, offering a promising solution to the industry's urgent need for exposure controls. Future field-based studies are needed to evaluate the effectiveness of the LEV system under a wide variety of workplace conditions, including varied work practices, facility sizes, and room ventilation conditions.

## 5 Conclusion

Exposure controls are urgently needed to mitigate potentially harmful inhalation exposures during cannabis processing activities. The partially-enclosed hood LEV system demonstrated strong reductions in PM concentrations during cannabis flower grinding and automated joint filling in a controlled laboratory setting. While field-deployed testing is necessary to fully assess the LEV system efficacy, these preliminary findings suggest that a partially-enclosed hood design effectively controls PM concentrations during cannabis production without compromising existing work practices.

## Data Availability

The original contributions presented in the study are included in the article/Supplementary material, further inquiries can be directed to the corresponding author.
