# Supplementary material for: Pilot study of a local exhaust ventilation system to control particle and terpene concentrations during cannabis processing
Source: Front Public Health. 2026 Oct 23;14:1964267. doi: 10.3389/fpubh.2026.1964267 (PMC13739690; doi:10.3389/fpubh.2026.1964267)
Supplement: Supplementary file 1 [file Data_Sheet_1.docx]

Supplementary Material

**Table S1: Summary of mass aerodynamic diameter size during grinding and pre-roll joint filling measured by APS**

|  | **Grinding** | **Pre-roll joint filling** |
| --- | --- | --- |
| **Median (µm)** | 3.17 | 3.82 |
| **GSD (µm)** | 1.87 | 2.00 |

Particle size was measured continuously by the aerodynamic particle sizer (APS) during one trial of grinding (Trial 2) and joint filling (Trial 2) without the local exhaust ventilation system. Density of 1 g/cc was assumed.

**Figure S1: Mass aerodynamic particle size distribution during cannabis grinding measured by APS**

Mass aerodynamic diameter distribution of particles during one cannabis grinding trial (Trial 2) without the local exhaust ventilation system. Particles were measured continuously using the aerodynamic particle sizer (APS) and assumed to have a density of 1 g/cc. Aerodynamic diameter was plotted on the log_10_ scale.

**Figure S2: Mass aerodynamic particle size distribution during pre-roll joint filling measured by APS**

Mass aerodynamic diameter distribution of particles during one cannabis joint filling trial (Trial 2) without the local exhaust ventilation system. Particles were measured using the aerodynamic particle sizer (APS) and assumed to have a density of 1 g/cc. Aerodynamic diameter was plotted on the log_10_ scale.

**Table S2: Terpenes with analytical reporting limits**

| **Terpene** | **Reporting limit (ng)** |
| --- | --- |
| D-Limonene | 80 |
| alpha-Pinene | 40 |
| beta-Pinene | 80 |
| Terpinolene | 40 |
| Linalool | 40 |
| Camphene | 40 |
| beta-Caryophyllene | 40 |
| beta-Myrcene | 40 |
| alpha-Humulene | 40 |
| Fenchol | 40 |
| p-Cymene | 40 |
| alpha-Terpinene | 40 |
| gamma-Terpinene | 40 |
| 3-Carene | 80 |
| alpha-Terpineol | 40 |
| Borneol | 100 |
| Eudesmol | 40 |
| alpha-Bisabolol | 40 |
| DL-Menthol | 100 |
| Nerolidol II | 40 |
| Citronellol | 100 |
| Cedrol | 40 |
| Fenchone | 40 |
| Nerolidol I | 40 |
| Camphor | 80 |
| alpha-Cedrene | 40 |
| Geraniol | 100 |
| Geranyl Acetate | 40 |
| Isoborneol | 100 |
| Phytol | 200 |
| Pulegone | 100 |
